# P07 QI Project: delays in the blood culture pathway at a cancer care centre in South Yorkshire

**DOI:** 10.1093/jacamr/dlaf118.014

**Published:** 2025-07-14

**Authors:** Kathryn Lachlan-James

**Affiliations:** The University of Sheffield Medical School, Sheffield, UK

## Abstract

**Background:**

In 2023–24, there were 489 123 cases of sepsis diagnosed in England. Timely collection and analysis of blood culture samples is vital to diagnose and treat these bacteraemias appropriately. The blood culture pathway advises that samples should be loaded for analysis within 4 h of collection. Significant delays were identified at Sheffield Teaching Hospitals (STH) sites.

**Objectives:**

This evaluation focuses on evaluating the delay between collection and loading for analysis at STH, specifically Weston Park Hospital, a specialist hospital in Sheffield dedicated to non-surgical cancer care. The project will evaluate the blood culture pathway at STH with an aim to raise awareness of the blood culture pathway and publicize the transport schedule in and out of hours.

**Methods:**

Questionnaires were used on clinical staff to assess current knowledge of the blood culture pathway and transport. The two wards with the highest output of blood cultures were selected. Data collection was between 20 January 2025 and 14 February 2025. Audit data were collected from the microbiology laboratories between 3 March 2024 and 2 March 2025. Discussions were initiated between ward clinical staff, microbiology doctors, and facilities regarding transport.

**Results:**

Gaps in knowledge were identified regarding laboratory location and sample drop-off location in and out of hours. Issues were identified in out of hours transport with impaired access due to security measures. Mean and median times between collection and loading were collected from audit data and analysed to show trends and proportions of samples arriving below the target time.

**Conclusions:**

The questionnaire data have identified rectifiable causes for delay. Posters were created to address misconceptions and inform clinical staff on transport availability and distributed via the clinical educator. Future work includes reassessing knowledge of the blood culture pathway and transport, ensuring posters are up to date, and advocating for the inclusion of information on the pathway and its importance in induction training for new staff. These findings are important for improving patient care and may help others in assessing reasons for delay in sample transport and analysis.

